# Parenting in Cerebral Palsy: Understanding the Perceived Challenges and Needs Faced by Parents of Elementary School Children

**DOI:** 10.3390/ijerph20053811

**Published:** 2023-02-21

**Authors:** Ana Guimarães, Armanda Pereira, André Oliveira, Sílvia Lopes, Ana Rita Nunes, Cleia Zanatta, Pedro Rosário

**Affiliations:** 1 Psychology Research Center, School of Psychology, University of Minho, 4710-057 Braga, Portugal; 2 Department of Education and Psychology, School of Human and Social Sciences, University of Trás-os-Montes and Alto Douro, 5000-801 Vila Real, Portugal; 3 School Psychology Office, Agrupamento de Escolas da Nazaré, 2450-284 Nazaré, Portugal; 4 Centro de Ciências da Saúde, Catholic University of Petrópolis, Petrópolis, Rio de Janeiro 25685-100, Brazil

**Keywords:** cerebral palsy, parenting, parenting challenges, parenting needs, periods of child development, family life contexts

## Abstract

Cerebral palsy (CP) can be considered the most frequent childhood physical disorder. The severity and type of dysfunction depend on the brain injury. Movement and posture are the most affected areas. CP is a lifelong condition, and parenting a child with this disorder brings additional challenges (e.g., dealing with grief) and needs (e.g., information). Identifying and characterizing their challenges and needs are pivotal to enrich the knowledge in this field and help draw more suitable support for parents. Interviews with 11 parents of children with CP attending elementary school were conducted. The discourse was transcribed, and a thematic analysis was performed. Three themes emerged from the data: (i) challenges of parenting a child with CP (e.g., internal challenges), (ii) crucial needs for parents to cope with a child with CP (e.g., information), and (iii) the intersection between challenges and needs of parents of children with CP (e.g., unawareness). Regarding the challenges and needs characterization, lifespan was the most frequent period of child development, and the microsystem was the context of life most reported. The findings may inform the design of educational and remediation interventions to support families of children with CP attending elementary school.

## 1. Introduction

Cerebral palsy (CP) can be considered the most frequent childhood physical disorder [1]. In Europe, the prevalence is 1.5 to 2.5 children per 1000 live births, and worldwide about 17 million people live with this disability [2,3]. CP is caused by an early brain injury and may occur during pre-, peri-, or postnatal periods [1,4,5]. The severity and type of dysfunction depend on the brain injury’s size, type, and location. Movement and posture are the most affected areas [5], and CP can be classified according to the nature of the motor impairment (i.e., primary impairment) and the parts of the body affected (topographic classification). Regarding the nature of the motor impairment, there are four types: dyskinetic (i.e., uncontrolled and writhing movements); ataxic (i.e., difficulties in coordination and balance); spastic (i.e., increased muscle tone); and mixed [6,7]. The topographic classification includes diplegia (i.e., lower limbs more affected than upper limbs); hemiplegia (i.e., upper and lower unilateral extremity impairment); and quadriplegia (i.e., four extremity impairment). Secondary impairments are prone to be associated with this disorder, such as intellectual disability, communication problems, epilepsy, behavior disorders, and bladder control problems [8,9]. Commonly, typically developed children achieve independence in basic self-care activities during middle childhood [10]. In contrast, children with CP are prone to self-care limitations and require specialized help to fulfill care needs [11]. Moreover, acknowledging that CP is a lifelong disorder, these children face a long-term dependence on the parent, which forces them to perform different roles and brings additional challenges and needs [5,12,13].

### 1.1. Parenting Experiences in CP: Challenges and Needs

Whittingham et al. [14] investigated the parenting challenges in raising children with CP through a focus group with parents and health professionals with expertise in CP intervention. Findings reported a set of parental challenges as follows: grief dealing (i.e., diagnosis and adjustment in the expectations), additional parenting tasks (e.g., help in mobility), and parenting under public scrutiny (i.e., stigma perception) [14]. The study by Alaee et al. [15] interviewed 17 parents of children with CP. Through parents’ perspectives, it was possible to identify two types of parental challenges: social (e.g., inadequate facilities and services) and psychoemotional (e.g., being worried) [15]. Finally, the review by Elangkovan and Shorey [16] analyzed the experiences and needs of parents caring for children with CP. Findings underlined the physical load related to the parents’ childcare tasks, such as bathing and toileting [16].

Along with research on parenting challenges, another line of research has analyzed over the last years the parent’s needs to cope with their children with CP. For example, Palisano et al. [17] have shown that more than 50% of the participants (501 parents of children and youth with CP) reported the need for information (e.g., plan the future) and the need to have time to themselves. In addition, Buran et al. [18] reported that parents with children with CP identified the need for services (e.g., recreational/entertainment) as the most relevant need, followed by the need for information (e.g., planning the future of the child).

More recently, Dieleman et al. [19] conducted research aiming to deepen our understanding of parental experiences while taking care of adolescents with CP. Grounded on the Self-Determination theoretical framework [20], these authors analyzed the parents’ basic psychological needs (i.e., the need for autonomy, relatedness, and competencies). Findings indicate that parents reported facing challenges related to the need for autonomy (e.g., at least one of the parents reported being unable to satisfy professional goals fully), need for relatedness (e.g., many parents reported to have declined an invitation to participate in a social activity not adapted to their child needs) and need for competence (e.g., difficulties to help children accept their disability). Moreover, Dieleman et al. [19] also reported that parents of children with CP are likely to experience difficulties accepting the diagnosis and dealing with the uncertainty related to the child’s future. This finding is consistent with Barreto et al. [21]’s work, suggesting depression and anxiety are more prevalent for parents of children with CP when compared with parents of children typically developed.

Altogether, findings suggest that the parental challenges and needs are multi-level (i.e., present in different systems), such as parents’ mental health, family routines, lack of information, competency, or access to services. For example, Majnemer et al. [22] underline that the challenges faced by children with CP and their families differ in each stage of child development. In another study, Hummelinck and Pollock [23] examined the needs of parents of a chronically ill child and concluded that their need for information varied over time. In sum, prior research mapped the challenges and needs of parents of children with CP. However, to the best of our knowledge, no studies have analyzed the challenges and needs of these families over child development conjointly. To achieve this purpose, one theoretical orientation and one theoretical framework informed our study: (a) The five periods of child development and [24] (b) Bronfenbrenner’s Ecological Systems Theory [25], respectively.

### 1.2. The Five Periods of Child Development

The division of lifespan development into periods is a social construction that helps to organize individuals’ development achievements and needs during their lives [24]. Despite the likeness of development progression across cultures, some acquisitions vary in children’s cultural specificities. Nevertheless, there seems to exist consensus regarding the skills acquisition occurring during middle childhood, adolescence, and adulthood upon foundational skills developed from the early stages of childhood [26]. The present study followed the sequence of the five (plus adulthood) periods generally accepted in Western societies. Each period considered the typical significant development acquisitions, considering human development’s physical, cognitive, and psychosocial domains. Specifically, the following periods were considered: prenatal (from conception to birth), infancy and toddlerhood period (birth to age 3), early childhood (ages 3 to 6), middle childhood (ages 6 to 11), adolescence (ages 11 to about 20), and adulthood (since age 21) [24].

The prenatal period is typically characterized by the most rapid physical growth during a lifetime, while body structure and organs are developed, highlighting the exponential growth of the brain. Furthermore, this phase emphasizes vulnerability to environmental influences during the interaction between genetics and environmental conditions. Regarding the cognitive domain, this period is marked by the ongoing development of abilities crucial to learning and remembering functions and the beginning of a sensory stimuli response. At this stage, children can respond to the mother’s voice (i.e., cognitive domain) while developing a rudimentary form of social interaction (i.e., psychosocial domain).

The infancy and toddlerhood period (birth to age 3) characterizes a period susceptible to environmental influence where the brain complexity grows. During this period, children are expected to show abilities to learn and remember (even in the early weeks), and by the end of the second year, they can use symbols and solve problems; their comprehension and use of language are in constant development. The attachment to parents and others is the psychosocial development marks of this period: the development of self-awareness, the shift from dependence to autonomy, and an increased interest in other children.

The stabilization of physical growth characterizes early childhood (ages 3 to 6). Typically, appetite diminishes, and sleep problems are common. During this period, handedness is clearly defined, and occurs an improvement in motor skills (fine and gross) and strength. Cognitive development is in progress. The children present slightly egocentric thinking; memory and language improve, and intelligence becomes more predictable.

Regarding psychosocial development, self-concept, and emotional understanding become more complex. Independence, initiative, and self-control increase during this period alongside gender identity development. Playing time becomes more imaginative and elaborate and typically more social. Altruism, aggression, and fearfulness are common.

In middle childhood (6 to 11), growth tends to slow progressively. This developmental period is particularly relevant since it marks the transition from kindergarten to formal education, i.e., the entrance into elementary school. There is an improvement in strength and athletic skills. Children begin to think logically but concretely, and memory and language skills increase; moreover, egocentrism is likely to diminish. Psychosocial development is characterized by a complexification in the self-concept, affecting self-esteem. Co-regulation reflects gradual shifts in control from parents to children. Peers assume central importance.

Adolescence (ages 11 to 20) is characterized by rapid and profound physical growth. Reproductive maturity occurs. It is a period with significant health risks resulting from behavioral issues, such as eating disorders and drug abuse. The cognitive development hallmark of this period is the ability to think abstractly and use scientific reasoning, while the search for identity (including sexual identity) is central to psychosocial development. During this period, peer groups may positively or negatively influence youth. Education focuses on preparation for college or vocational paths.

Children with developmental disabilities or medical conditions (e.g., CP, epilepsy) are likely to follow a distinct developmental journey regarding the pace, goals, and achievements of typical development children. Commonly, non-typical developing children suffer from delays and impairments in different areas [27]. For example, the ability to walk is typically acquired between nine and 17 months (an average of 11 months), but for children with CP, the average age to gain this skill is 19.61 months (a delay of eight months to develop this skill) [28,29].

### 1.3. Bronfenbrenner’s Ecological Systems Theory

According to Bronfenbrenner [25], understanding human development is a complex process that “requires examination of multiperson systems of interaction not limited to a single setting and must take into account aspects of the environment beyond the immediate situation containing the subject” (p. 514). This theoretical framework helps to understand the interconnections between people and the environment through four levels: micro-, meso-, exo-, and macro-systems. The microsystem refers to interactions between one or more individuals and the environment (e.g., parent, teacher). At this level, parenting practices and parent-child interaction are essential in educating a child with a disability [30]. Mesosystem encompasses relationships and connections between two or more microsystems. For example, the caregiver’s marital relationship (microsystem) is expected to influence the parent-child relationship (microsystem), impacting children’s life [30]. The exosystem is an extension of the mesosystem and includes the major institutions of society (e.g., governmental agencies). At the same time, the macrosystem comprises the values, laws, and cultural mores framing individual life (i.e., culture). Importantly, this level of analysis is likely to influence the other three systems of the model [25]. For example, the healthcare delivery system is particularly relevant for parenting a child with a disability [30]. For this study, we explored three systems (micro-, meso-, and macro).

### 1.4. Study Purpose

Caregiving a child with CP is a lifelong quest [5]. This educational journey has many challenges and needs very distinct from those expected in the parenthood of a typically developed child. These parents are likely to show poorer psychological well-being than parents of children with typical development and lower levels of quality of life when compared to the general population [21,31]. These findings are significant because, as Rosenbaum [32] warns, children’s well-being is likely influenced by their parents and family’s well-being. However, caring for a child with CP demands a constant adaptive process, and families struggle to balance and respond to their children’s needs [16]. Therefore, further understanding these parents’ challenges and needs is pivotal. Grounded on this urge, the present study aims: (i) to describe the perceived challenges and needs (period[s] of child development and context[s]) of parents of children with CP attending elementary school; (ii) to understand further the relationships between the challenges and needs identified. The second goal emerged during the data collection and analysis processes. Hopefully, the information gathered in this study will enrich the knowledge in this field and help draw more suitable support for parents’ challenges and needs with an expected impact on enhancing the family’s quality of life.

## 2. Materials and Methods

### 2.1. Study Design

The study followed a qualitative descriptive methodology. This design is commonly used when the study purpose is to capture the experiences of individuals to better understand a phenomenon or event [33,34]. According to Sandelowski [34], the qualitative descriptive methodology is the most appropriate when researchers want to gather information that can help develop interventions or policies, as in this study’s case. A multiple case study approach was selected. This approach is used when the purpose is to generate an in-depth, multi-faceted understanding of a complex issue in its real-life context. The multiple case study involves studying various cases (simultaneously or sequentially) to develop an even more extensive appreciation of a particular issue [35].

Two research questions guided our study: (i) What are the perceived challenges and needs faced by parents of children with CP attending elementary school?; (ii) When (five periods of child development) and in which context (Bronfenbrenner’s Ecological Systems Theory) do these perceived challenges and needs occur? A new research question emerged during the data collection and analysis process: (iii) What are the associations/links between the perceived challenges and needs?

### 2.2. Participants and setting

This study sampling follows Robinson’s [36] four-point approach to qualitative sampling: sample universe, sample size, sampling strategy, and sample sourcing. Two inclusion criteria were considered: (i) be a parent of a child diagnosed with CP; (ii) the child attends elementary school. No exclusion criteria were applied. The research team defined a sample size range between 9 and 17 participants. This decision was made based on Hennink and Kaiser’s [37] work, according to which it takes 9 to 17 interviews to reach data saturation. The recruitment process occurred in two rehabilitation centers in the north of Portugal. From all parents that fit the inclusion criteria, the therapists helped the research team to identify the best informants for the study.

A total of 12 parents were invited to participate in the study. Eleven decided to participate. One mother did not agree to participate due to fatigue resulting from a recent participation in a research study. This paper reports findings drawn from 11 parents (nine mothers and two fathers), aged between 25 and 49 years (*M* = 39.55, *SD* = 6.65). More than 50% of the participants had completed high school or college. Most participants have more than one child (cf., Table 1). One of the mothers has two children (twins) that fit the inclusion criteria (P4). Regarding participants’ children, nine were male (*N* = 12), aged between seven and ten years (*M* = 9.08, *SD* = 1.56). All 12 children were attending a mainstream public school. The Portuguese educational system is organized accordingly to the multi-tiered system of support. The multi-tiered system of support provides a method of identification and intervention to support students with difficulties (temporary or permanent) in their learning process. The support is offered to scaffold the student’s needs. There are three tiers of cumulative support: tier (1) universal or primary (e.g., academic small group intervention); tier (2) secondary (e.g., mentoring support); and tier (3) tertiary (e.g., significant curricular adaptations) [38]. Seven children were benefiting from support foreseen in tier (2), and five from support foreseen in tier (3) (cf., Table 2).

### 2.3. Data Collection

The data collection design followed three phases. First, therapists provided information about the children’s clinical condition. The children’s Gross Motor Function Classification System level (I to V), indicating the motor severity impairment, was among the information collected [39]. Second, just before the interview, participants were asked to fulfill a sociodemographic questionnaire (e.g., literacy level, number of children) and to visualize an educational video (cf., Section 2.3.1. Educational video). Third, the participants responded to the semi-structured interview.

#### 2.3.1. Educational Video

Participants were invited to visualize a five-minute educational video (translated to Portuguese in audio and using subtitles). A father of a child with CP created the video [40]. The video presents the expectations, difficulties, and adaptations to the fatherhood of a child diagnosed with CP, namely, the diagnosis impact and acceptance and the parenthood expectations management and adjustment (e.g., acceptance, guilt feelings, setting priorities). In the current study, this video was used for an ice-breaker purpose. This option was grounded on the fact that parents of children with CP are frequently questioned about their children’s developmental goals and difficulties felt in the process. Consequently, their narrative is likely to follow a practical and goal-driven approach. Through the presentation of a peer experience, we aimed to promote an emotional connection with characters and instigate participants’ readiness to reflect on the challenges and needs of parenting children with CP [41]. The rationale for using the video was theoretically rooted in the model by Larkey and Hecht [42]. These authors conceptualized a theoretical model (Narrative as Culture—Centric Health Promotion) to describe the relationships between the narrative and their effects on health behaviors. This model has three levels: (i) the narrative characteristics (i.e., related to the involvement of the person exposed to the narrative with the characters, the story, and the cultural aspects associated); (ii) the mediators (i.e., engaging people who are watching the video with the story on a personal level); and (iii) the outcomes/responses (i.e., transferring the lessons learned on the narrative to the real world). The three levels affect each other sequentially. The main persuasive effects identified by this model are emotional connection (i.e., empathy and linking) and identification with the characters [41,43]. The video used in this study matched the narrative characteristics mentioned in the model as follows: realistic (a nearby store), emotional connection, and identification with the characters (“that person is like me”) [42].

#### 2.3.2. Semi-Structured Interview

After the visualization of the video, parents participated in a semi-structured interview. This qualitative data collection method uses open-ended questions to promote detailed storytelling sharing about participants’ perceptions [44]. The interview script included six open-ended questions to (i) explore participants’ feelings and thoughts while watching the educational video (e.g., How did you feel watching this video? What do you think about the message conveyed?); and (ii) learn their thoughts about being a parent of a child with a developmental disorder (e.g., What it is like to be a father of a child with a developmental disorder?). Data collection occurred in a soundproof room at the rehabilitation centers (i.e., the medical office). Each interview lasted between 16 and 74 min. After nine interviews, no new themes emerged, indicating saturation was reached [45].

### 2.4. Data Analysis

The interviews were analyzed through thematic analyses [46,47]. Before starting an in-depth analysis, a coding frame (i.e., codebook) was created. The codebook was based on: (i) the theoretical background in parenting experiences in CP [14,17,19]; (ii) the five periods of child development [24]; and (iii) Bronfenbrenner’s Ecological Systems Theory [25] (deductive approach). It contained the name of each code and a definition. The data analysis followed the six-phase described by Braun and Clarke: (i) familiarization with the data; (ii) coding; (iii) searching for themes; (iv) reviewing themes; (v) defining and naming themes; and (vi) writing up [46,47]. News codes emerged from the data during the codification process and were added to the codebook (inductive approach). Of the five periods of child development, only the first four were considered in the codification process. The interviewed parents were not experienced as caregivers of adolescent or adult children (development period of adolescence/adulthood). Therefore, these periods have been included in a more comprehensive code termed “lifespan.” Data analysis was conducted with the assistance of QSR International’s NVivo 10 [48] software for Windows^®^ (Burlington, MA, USA). This software simplifies the data analytics process and provides tools for and support in the search process of map interconnections between codes and checking for patterns [49]. The codification of the data involved two researchers (A.G. and A.P.) with training in qualitative analysis to ensure trustworthiness to the process. A.G. codified all the data, and A.P. codified 30%. A consensus was reached after discussing the discrepancies in the codification. According to Landis and Koch, the inter-observer agreement reached (*K* = 0.90), indicating an almost perfect agreement [50].

### 2.5. Ethics

The study was carried out under the recommendations of the Ethics Committee for Research in Social and Human Sciences of the Ethics Council of the University of Minho (CEICSH 068/2019). All participants gave written consent following the Declaration of Helsinki and authorized the collection of information about their children’s medical diagnoses with the therapists. The interviews were audio-recorded and transcribed verbatim. Participation was voluntary and unrewarded.

## 3. Results

Three themes emerged from the data: (1) challenges of parenting a child with CP, (2) crucial needs for parents to cope with a child with CP, and (3) the intersection between challenges and needs of parents of children with CP (cf., Table 3). The challenges and needs were categorized regarding the period(s) of child development (i.e., prenatal, infancy and toddlerhood, early childhood, middle childhood, and lifespan periods) and the context(s) (i.e., micro-, meso-, macro-, systems) in which they occur. The results report followed the theme’s order. Participants’ verbatim quotes were introduced to illustrate the categories and patterns identified.

### 3.1. Challenges of Parenting a Child with CP

According to participants’ speeches, parenting a child with CP is not a smooth hike. It is more like a climb: full of ups and downs but primarily ongoing challenges. During the analysis of the interviews, we identified three types of frequent, ongoing challenges: (i) daily care challenges, such as additional tasks, family management, and comprehension of children’s functionality, (ii) internal challenges, such as diagnosis acceptance, mental health issues, and achievements, and (iii) social challenges, such as comparison, and stigma. The three challenges were defined and characterized as follows:

#### 3.1.1. Daily Care Challenges

Participants reported daily care challenges, i.e., demanding situations common in parental routines to take care and educate a child with CP, and seem to understand these situations as life-long challenges [lifespan]. The child’s clinical condition frequently forces parents to perform additional parenting tasks, such as attending therapies with their children or supporting them in therapy exercises at home. For example, hygiene/food continuous care inconsistent with that provided for a child with a typical development with similar age (e.g., use diapers at seven years old; micro-system) is also frequent, as illustrated by parent 10 (P10):

P10: *I need to take care of her hygiene every day. B. is totally dependent. I need to do everything for her. Dress her, give her food, bathe her, change her diapers, and put her to poop.*

The additional parenting tasks, combined with strict time management and the willingness to support the child as much as possible, lead parents to adjust family dynamics. The participants reported job adaptations such as working part-time or being a full-time parent [meso-system]:

P5: *My husband and I agreed that I needed to quit my job. I stay at home taking care of our daughter and doing everything for her benefit rather than earning money or having a career.*

The complexity of the ongoing care and educational challenges often leads to establishing a parent-child bond described as strong. Parents get to know and comprehend their children deeply. Some of them, as parent 8 (P8), reported the ability to anticipate the child’s wishes or needs:

P8: *[…] G. cannot communicate clearly, and it isn’t easy to follow him. So, to communicate with him, sometimes we must guess his possible needs or wishes. […] We must put ourselves in his shoes and anticipate the word he wants to say […].*

#### 3.1.2. Internal Challenges

Parents reported feeling several internal challenges, i.e., internal struggles related to being a parent of a child with CP [micro-system]. For example, the diagnosis moment was described as an instant of anger, guilt, grief, and perceived unfairness. For some parents, the acceptance process was completed during the child’s first years [infancy and toddlerhood], but for others, it is still a work in progress [lifespan]. Parent 11 (P11) quotation illustrates the complexity of this process.

P11: *At home, A. is entirely accepted, but when you learn the diagnosis, at that very moment it isn’t easy to accept. It is a shock, and the perspective of the future is terrifying.*

Factors such as the diagnosis, the child’s specific needs, the worries related to the abilities to cope with the condition demands, and the efforts needed to provide the child with the best quality of life lead parents to deal with mental health issues continuously. Participants reported mental exhaustion and breakout moments. Mental health issues seem to be perceived as a challenge to deal with during the child’s life [lifespan]. The following quotation from parent 10 (P10) illustrates this experience of exhaustion:

P10: *There were days… I just wanted to disappear. These days still exist. Believe me.*

After diagnosing CP as a long-term health condition, parents’ expectations about their child’s abilities are limited. Therefore, each achievement, despite being small, was described as a hallmark for the family’s hope increasing [lifespan], as illustrated by parent 9 (P9):

P9: *We did not expect that he would walk, and today he walks; G. was not expected to succeed in school, but he attends school and has good grades.*

#### 3.1.3. Social Challenges

Findings suggest the existence of social challenges, i.e., comparison, stigma, and exclusion situations occurring during the child’s development [lifespan]. Parents understand the comparison of child and parenting related as unavoidable through the child’s development [lifespan]. The comparisons with children with typical development were more frequent within the family context, i.e., between the child with CP and a sibling [micro-system]. Typically, the comparison with other children with disabilities (peers) occurred in rehabilitation centers or hospitals [micro-system]. Some comparisons and commentaries about specific parental practices were also reported [meso-system]. The following quote illustrates a comparison of the ability to eat breakfast independently between a child with CP and his typically developed sisters.

P5: *M. cannot eat by herself. For example, I must be there for her at breakfast and give her the food. With her siblings is an entirely different situation. They are independent.*

In addition, parents reported a persistent societal difficulty in accepting children with CP [macro-system]. The stigma situations were reported as pointless, very sad, and, in some cases, somehow traumatic. Children’s social interactions, such as ordering something at a cafeteria, were reported as very stressful for parents, as described by P10:

P10: *I need to make considerable efforts to keep calm, not be upset and ignore the people staring at my child and me.*

### 3.2. Crucial Needs for Parents to Cope with a Child with CP

The parents’ permanent need to learn more about their children’s clinical condition, how to find or be supported, and how to keep themselves healthy and able to the parenthood struggles never seem to end. Analysis of the interviews allows the identification of three frequent areas of need, such as information, support, and personal well-being. These three areas of need are characterized as follows.

#### 3.2.1. Need for Information

Need for information refers to a lack of knowledge about the child’s clinical condition and its implications. Information is crucial to better understand and make adjustments to the child’s needs. Findings suggest that the need for information was transversal to the previous and present periods of the child’s development. The lack of knowledge reported by the parents concerned themselves and other contexts in the child’s life. Furthermore, parents seem to project that this information need will continue in the near and not-so-near future [lifespan]. Regarding the first, parents reported feeling the need to constantly update their information about the disability (in itself) and parenting skills (e.g., the process of taking care and educating a child with a long-term health condition, the rehabilitation process, the prognostics, and the child/family plans) [micro-system], as illustrated by parent 4 (P4):

P4: *I am always searching for new strategies to help my kid communicate more.*

The parents reported that the child and family surrounding contexts, such as social, educational, or health institutions (e.g., social services, hospitals, and schools), are frequently poorly informed about the specificities of CP or even unaware of these characteristics. For example, a mother mentioned that the typical food meal offered in schools to children with CP with feeding impairments does not meet their needs or follow the medical recommendations [meso-system]. The last context mentioned was the “society as a whole” [macro-system]. Parents seem to feel that society cannot cope well with children with disabilities. Consequently, children with CP are more prone to face a strong challenge: ‘the stigma of being different.’ Parents reported that some children with CP struggle to find peers available to play in the playground or adults open to listening to their needs without judging. The following quotation demonstrates a stigma situation anticipation:

P11: *For a long time, I didn’t let my son order in cafes or stores because I knew they would look at him like an alien [...]. We don’t have a society prepared to understand these kids.*

#### 3.2.2. Need for Support

The need for support refers to the urge to be comforted, heard, and share thoughts, doubts, and insecurities. In other words, be accompanied by someone with knowledge or experience about the situation [meso-system]. This need to be supported, which the parents pointed out as a past, present, and (most certainly) future need [lifespan] seems to have an intensity peak just after the child’s birth [infancy and toddlerhood]. Usually, it is during this time frame that the diagnosis is known. This time window is typically characterized as a time of anxiety, anger, and loss in which accompaniment, or its absence, was noted as very important. Talking with parents of older children with CP (e.g., in the waiting room of the rehabilitation center) was reported as a positive experience. Parents understood these conversations as opportunities to gather information (e.g., feeding tips, expectation management) and benefited from peers’ support (e.g., a better understanding of new possibilities and alternative paths to design their family routines), as illustrated by P11:

P11: *I learned much in the waiting rooms while sharing experiences with other parents; sometimes, these moments were more important than those spent in the therapies.*

Another topic mentioned was the need for support from a formal caregiver who could take care of the child for some time. This support would allow parents to have time for themselves (e.g., go to medical appointments, rest in the coach, take a leisurely day). Some participants also reported feeling the need to share and support other parents at the beginning of their experience caring for a child with CP. Although each person has their process to follow, these informal and circumstantial mentors have experienced in-depth the anxiety and the confusion that characterizes the first moments and are willing to make this process more accessible for other parents:

P9: *What is most important is to share our experiences and knowledge [...]. Transmit to these parents diagnosed a short time ago, and typically very anxious about the future, that the world does not end there and that everything is possible, depending on the parent’s beliefs.*

#### 3.2.3. Need for Personal Well-Being

Well-being can be described as a balance between the individual’s resources and the challenges they have to face [51]. CP is a life-long condition demanding continued care over the child’s development [lifespan/micro-system]. Through participants’ experience, caring for and educating a child with CP was pictured as emotionally and physically demanding, as the following quote illustrates.

P5: *I feel tired, very tired. I’m struggling with my back, soon M. will be in physiotherapy, and so will her mother. Because picking up M. two, three, four times a day is exhausting [...]. She’s big, she’s not heavy, but she’s big. And picking up is tiring.*

Besides, parents revealed a strong commitment and daily effort to improve their child’s lives: however, the health developments are not always consistent with the efforts displayed. As parent 2 (P2) illustrates, the demoralization after an unsuccessful attempt, the slow improvement process, and the limited control over the results seem to impact the parents’ well-being.

P2: *It wasn’t until recently that I became very upset with the idea that he was not walking. And that [the child being able to walk] for a few years was my daily struggle. Always running from here to there to help him to be able to walk independently, and now I’m starting to realize that it’s being difficult, and it’s starting to hit me harder. […] make us know that it would be more complex than we had imagined in all these years.*

### 3.3. The Intersection between Challenges and Needs of Parents of Children with CP

The challenges related to parenting a child with CP and the needs related to the parenthood demand are closely related. From the analysis of the interviews, we identified four frequent types of challenges and needs co-occurrent: task-related information, unawareness, diagnosis acceptance support, and balance. The four subthemes will be described below.

#### 3.3.1. Task-Related Information [Daily Care Challenges X Need for Information]

Being a parent of a child with CP seems to be a continuous learning process. Findings suggest that the specialized tasks parents must perform (e.g., going to suitable therapies and preparing adequate food) lead to a frequent search and acquisition of new knowledge:

P11: *[…] Fifteen days after my baby left the hospital, he started physical therapy. I found it so strange; I was confused because I didn’t understand the purpose. He was just a little baby.*

#### 3.3.2. Unawareness [Social Challenges X Need for Information]

Parents reported that despite feeling the stigma situations as inconvenient, they perceived most of them as unintended. From their perspectives, these situations seem more associated with a lack of knowledge and awareness about the child’s disability, as illustrated by P10.

P10: *My daughter started yelling in the supermarket once, and one lady covered his son’s ears. This child told her he wanted to yell too, but the mother replied: “No, son, you are not a freak.” That comment hurt me, hurt me deeply.*

#### 3.3.3. Diagnosis Acceptance Support [Internal Challenges X Need for Support]

Findings indicate the relevance of parents having access to support immediately after the diagnosis and during the acceptance process. Parents seem to need someone to help them cope with the diagnosis and answer their questions, as suggested by parent 3 (P3). Participants suggested educators with proper training in these situations and parents of older children with CP as possible supporters.

P3: *[…] I think parents must have some support at the beginning (a short period after the diagnosis). They need to have someone, an office… where to go and get help.*

#### 3.3.4. Balance [Internal Challenges X Need for Personal Well-Being]

Findings suggest that parents’ mental health is affected by the mentally exhausting and arduous task of taking care and educating a child with CP. Sometimes, this demand makes parents forget about themselves. The need to give up pleasant activities (e.g., playing football with friends) to be present for their children was also reported, as demonstrated in the following quotation of parent 7 (P7):

P7: *[…] As parents, we always give up on many things because of our children. That is the way. […] When we talk about a child with a clinical condition [such as CP], the things we need to give up are double or triple.*

## 4. Discussion

The present study aimed to understand the process of parenting an elementary school child diagnosed with CP. More specifically, identify the perceived challenges and needs faced by the parents and the relations between them. Furthermore, we intended to characterize the challenges and needs accordingly to the period(s) of child development (e.g., prenatal) and the context(s) (e.g., microsystem) in which they occur.

### 4.1. Parenting Challenges and Needs Identification

Three themes were identified: (1) the challenges of parenting a child with CP, (2) the crucial needs for parents to cope with a child with CP, and (3) the intersection between the challenges and needs of parents of children with CP. The first is consistent with results found in previous studies reporting that parents of children with CP experience particular parenting challenges while coping with their children’s clinical condition demands [52,53]. In the present study, participants reported three major challenges: daily care, internal, and social. Regarding daily care, challenges refer to topics such as the additional and not expected tasks parents must perform (e.g., conducting therapy exercises at home or feeding a 10-year-old child) compared to those typical while parenting a child of similar age with typical development. These findings are consistent with the parental challenge “Additional daily parenting tasks” found by Whittingham et al. in 2011 [14]. Along with the everyday challenges come internal and social challenges. The internal challenges refer to topics related to parents’ mental health. Parents of children with CP are at an increased risk of experiencing high stress, anxiety, and depression [54]. Among the social challenges, stigma was the issue most referred to. These data are consistent with previous literature indicating that experiences of stigma and discrimination are frequent for children with disabilities and their families [55,56,57].

During the process, parents get involved in crucial needs (second theme). Findings indicate the presence of three types of needs: information, support, and personal well-being. The need for information was mainly related to topics about the child’s clinical condition (e.g., therapies and prognostics). In 2009, Buran and colleagues found several parental information needs topics, such as services available in the community (e.g., rehabilitation centers) and emotional/physical growth and development [18]. These findings are consistent with those found in the present study. As suggested in the literature, the need for support was also frequently reported by the participants [17]. An interesting finding on this topic was the need to support, particularly when the diagnosis is known. Parents are expected to be overwhelmed with emotions and doubts that need to be appeased. Acknowledging the distress felt by parents with a newborn with CP, particularly in the first times, some parents reported their willingness to help parents at the beginning of the parenting process. Lastly, current data stressed the need for personal well-being, but this finding was not commonly reported in previous studies. However, it is assumed that parents of children with developmental disabilities are more vulnerable to developing mental health problems and more likely to show dissatisfaction with life [58].

Resulting from the analysis of the interviews, the last theme comprises the participants’ frequent articulation between challenges and needs. A challenge seems to appear related to a need when the parents think they have not developed all the necessary strategies to face it. For example, one of the associations found was the “Diagnosis acceptance support,” which relates an internal challenge (i.e., diagnosis acceptance) with the need for support. As reported in the literature, parents in this study seem to see the search for support as a mechanism to cope with the child’s CP diagnosis [59].

### 4.2. Parenting Challenges and Needs Characterization

The lifespan period was the most frequently identified. Parents report most of the challenges and needs following the child’s growth, stressing experiences lived in the past and present and sometimes anticipating the future. This lifespan perspective is consistent with the work by Majnemer et al. [22], stressing that every period of child development brings new challenges. Interestingly, some challenges associated with independence building and the transition to adulthood have not been widely reported by participants [14]. Not surprisingly, the latter may indicate that the participants were more focused on the challenges proximal to the children’s age. Congruently, the challenge of transitioning to adulthood seems to be associated with the early adolescence period [60]. Considering the children’s age range, this challenge could still be distant in the participants’ time horizon. Current data are consistent with the work by Kruijsen-Terpstra et al. [61], suggesting that parents need different types of information over time. For example, after the diagnosis, parents seem to be more eager for information related to the causes of the disorder and possible impairments; in the middle childhood period (the developmental stage of almost all the participating children), these needs seem more related to the therapies and prognostics. The need for information always exists in these parents’ lives; however, the focus is on permanent change along with children’s development.

Challenges and needs do not occur in a vacuum. Parents and children navigate this process in interaction with different social contexts. Interestingly, in the present study, participants were mostly focused on micro-system-identified experiences. The microsystem is where the child receives the basic care crucial for development and embraces close relationships such as the ones with teachers, peers, caregivers, and parents. Parenting practices and parent-child interaction are part of this system [30]. Many of the challenges and needs identified by participants were relative to parent-child interactions. Therefore, the meso- (relationships between parents and schools or parents and healthcare institutions), but also the macro-systems (e.g., governmental agencies) may consider analyzing the voices of parents of children with CP. These findings, despite being preliminary, present an organized chart of parents’ challenges, needs, and relationships that is likely to help educators and physical and occupational therapists in their work with the families. Current findings may also support advocacy efforts, for example, among the ministries of education and health, for the cause of families with children with CP.

### 4.3. Limitations and Future Implications

Despite the promising results, some limitations must be considered and addressed in future studies. First, the qualitative approach allows an in-depth understanding of the phenomena; however, it prevents findings from generalizations. Therefore, results should be framed within the study’s participants’ perspective. Second, only one of the child’s parents was interviewed. Future research should consider interviewing both parents and complementing parents’ perceptions, collecting data along with other relevant informants (e.g., child grandparents). Thus, different methods of data collection (e.g., focus groups) should be considered. Third, all the participants’ children attend CP rehabilitation centers, where a family-centered approach is followed. It would be interesting to interview parents whose children are not in CP rehabilitation centers or parents whose children are in CP rehabilitation centers with different approaches to compare findings. The methodological option of video presentation appears to increase participants’ predisposition to talk about their parenting experiences. Parents reported identification with the story and its characters—“It looks like I was watching the story of my life” (Parent 9). This finding may be relevant for future research focused on a population with very little available time and exhausted by being systematically asked to talk about their children’s clinical condition, such as the case of parents of children with CP. Lastly, previous studies suggested that the number and types of needs expressed differ from family to family. For example, Almasri et al. [62] pointed out some predictors of needs for families of children with CP, such as child characteristics (e.g., Gross Motor Function Classification System, communication problems); family characteristics (e.g., social integration, income); and services characteristics (e.g., enabling and partnership, respectful and supportive care). Future research might consider including a larger sample to further analyze, compare, and contrast the abovementioned predictors.

## 5. Conclusions

In summary, the interviews allowed the identification of three major challenges, i.e., daily care, internal and social, and three needs, i.e., information, support, and personal well-being in the task of parenting children with CP. Four frequent challenge-need co-occurrences were identified: task-related information, unawareness, diagnosis acceptance support, and balance. Globally, these findings may provide a corpus of knowledge likely to inform the design of educational and remediation interventions to support families of children with CP attending elementary school.

## Figures and Tables

**Table 1:** Demographic characteristics of participants (*N* = 11).

Participant	Characteristics			
	Age	Gender	Educational Level	Number of Children
P1	40	Female	Higher education	1
P2	40	Female	High school	2
P3	40	Female	High school	2
P4	42	Female	Higher education	3
P5	38	Female	Elementary school	3
P6	25	Female	High school	1
P7	32	Female	Middle school	3
P8	40	Male	High school	2
P9	47	Male	High school	2
P10	42	Female	Elementary school	4
P11	49	Female	Middle school	1

**Table 2:** Demographic characteristics of participants’ children (*N* = 12).

Participants’ Child	Characteristics						
	Age	Gender	Type of CP	Quality of Tonus	Comorbidities	GMFCS *	Tier Level of Support
PC1	7	Female	Right hemiplegia	Without classification	West syndrome	I	2
PC2	8	Male	Tetra paresis	Spastic	---	IV	2
PC3	8	Male	Tetra paresis	Spastic	---	V	2
PC4	11	Male	Tetra paresis	Spastic	---	V	3
	11	Male	Without classification	Without classification	---	II	3
PC5	11	Female	Tetra paresis	Spastic	---	V	3
PC6	7	Male	Without classification	Spastic	---	IV	2
PC7	10	Male	Tetra paresis	Spastic	---	V	3
PC8	8	Male	Without classification	Without classification	---	II	2
PC9	10	Male	Without classification	Without classification	DiGeorge syndrome	II	2
PC10	8	Female	Tetra paresis	Without classification	---	III	3
PC11	10	Male	Without classification	Without classification	---	I	2

* GMFCS = Gross Motor Function Classification System [39].

**Table 3:** Themes, subthemes, and frequency of report in the 11 interviews.

Theme	Subtheme	f
Challenges of parenting a child with CP	Daily care	11/11
	Internal	11/11
	Social	11/11
Crucial needs for parents to cope with a child with CP	Information	11/11
	Support	11/11
	Parental well-being	10/11
The intersection between challenges and needs of parents of children with CP	Task-related information	6/11
	Unawareness	7/11
	Diagnosis acceptance support	7/11
	Balance	7/11

## Data Availability

Data is available from the first author upon reasonable request.
